# Vegetation growth and landscape genetics of *Tillandsia* lomas at their dry limits in the Atacama Desert show fine‐scale response to environmental parameters

**DOI:** 10.1002/ece3.6924

**Published:** 2020-10-28

**Authors:** Marcus A. Koch, Clara Stock, Dorothea Kleinpeter, Camilo del Río, Pablo Osses, Felix F. Merklinger, Dietmar Quandt, Alexander Siegmund

**Affiliations:** ^1^ Centre for Organismal Studies Heidelberg University Heidelberg Germany; ^2^ Heidelberg Center for the Environment (HCE) Heidelberg University Heidelberg Germany; ^3^ Instituto de Geografía Pontificia Universidad Católica de Chile Santiago de Chile Chile; ^4^ Centro UC Desierto de Atacama Pontificia Universidad Católica de Chile Santiago Chile; ^5^ Nees‐Institute for Biodiversity of Plants (NEES) University of Bonn Bonn Germany; ^6^ Department of Geography – Research Group for Earth Observation (^r^geo) Heidelberg University of Education Heidelberg Germany

**Keywords:** Atacama Desert, Chile, genetic diversity, hyperaridity, local adaptation, loma vegetation, Tillandsia

## Abstract

1. Ecosystem dry limits have been studied in the context of species biology, fitness, and interactions with biotic and abiotic parameters, but the interactive effects of these parameters remain underexplored. Therefore, information on the putative effects of global climate change on these ecosystems is often lacking.
2. We analyzed the interplay between fine‐scale landscape genetics and biotic and abiotic factors of terrestrial *Tillandsia* lomas in the hyperarid Atacama Desert, characterized by a fog‐dependent vegetation type almost entirely dominated by one single vascular plant species.
3. We showed that metapopulations of *Tillandsia landbeckii* are genetically connected over many hundreds of square kilometers, and despite having a large potential for clonal propagation, genetic diversity is regionally and locally structured. At the landscape level, genetic diversity correlates well with fitness parameters such as growth, flowering, and vegetation density. We also observed fine‐scale correlation with a 3‐D landscape model indicating a positive feedback with seasonal fog occurrence and availability. The various interactions of biotic and abiotic factors resulted in regular linear banding patterns of vegetation arranged orthogonally toward the landscape slope. Ex situ growth experiments indicated that *T. landbeckii* grows at optimal rates in this extreme hyperarid environment, and we can extrapolate mean biomass production for this ecosystem.
4. *Synthesis*. Our results suggest that the unique ecosystem of terrestrial *Tillandsia* lomas in the hyperarid Atacama Desert is an evolutionarily balanced and fine‐scaled system. The vegetation itself is composed of long‐lived and persistent modules. We developed a descriptive model of the various interacting factors, thereby also highlighting the severe threat caused by global climate change potentially associated with fog disturbance patterns along the Chilean Pacific coast.

## Graphical abstract

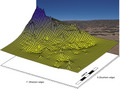

## 1 INTRODUCTION

Hyperarid regions are found all over the world and are home to unique ecosystems. Major hyperarid areas occur in Africa along the central and northern Sahara and the central Namib in Namibia; in Asia in the Rub' al Khali on the eastern Arabian Peninsula, the Iran/Afghanistan/Pakistan border area, the Taklamakan Desert in northwest China, and the Gobi Desert in Mongolia and China; and in southern America in the Atacama Desert (Huang et al., 2016). In these hyperarid areas, mean annual precipitation (MAP) is <25 mm (Thomas, 2011), and the aridity index, defined as the ratio of MAP to annual potential evapotranspiration, is <0.05 (Middleton & Thomas, 1997). The respective soil systems are among the harshest terrestrial biomes on Earth (Frossard et al., 2015) with surface temperatures ranging from 0 to 50°C (Eckardt et al., 2013; Fonseca et al., 2019). Water is extremely rare, and availability is often highly stochastic (Kumar et al., 2015).

The core of the Atacama Desert exhibited these extreme conditions together with interspersed episodes of enhanced fluvial activity during the Miocene and Pliocene but with a predominantly arid/hyperarid climate existing since the Early Miocene (Ritter, Stuart, et al., 2018). Thus, the Atacama Desert is one of the oldest hyperarid areas globally alongside the Namib (Eckardt & Spiro, 1999). The evolution of the entire desert system and its surface was predominantly driven by water‐limited processes thereby preserving extensive areas of fossilized landscape (Dunai et al., 2005; Rech et al., 2019; Ritter, Binnie, et al., 2018; Ritter, Stuart, et al., 2018). As a consequence, terrestrial life co‐evolved with the landscape and its water‐limited processes over millions of years (Böhnert et al., 2019; Knief et al., 2020; Mörchen et al., 2019; Zúñiga‐Reinoso & Predel, 2019).

Of biological interest is the coastal zone of northern Chile and southern Peru ranging from ~18°S to 30°S. Here, the ecosystems of the Atacama Desert stretch for ~1,300 km from sea level to 3,000 m a.s.l, and biotic components mostly depend on coastal fog. Vegetation occurs in disjunct patches, which are called lomas or fog oases (Cereceda et al., 1999; Rauh, 1958), and may be considered evolutionary remnants. Vascular plant species richness is high, and most of the 400–550 Atacama Desert species in Chile are endemic and threatened (Dillon & Hoffmann, 1997; Schulz, 2009). The adjacent Peruvian loma contain even more species (~850) owing to the higher water supply (Dillon et al., 2011). Both areas represent hot spots of biodiversity of high conservation value (Zizka et al., 2009). Evolutionary history is well reflected with high phylogenetic diversity, because only a few species from a given genus or family are extant (Heibl & Renner, 2012), which thereby increases overall average phylogenetic distance among taxonomic clades (Scherson et al., 2017).

Among the unique ecosystems and vegetation types in the Atacama Desert are *Tillandsia* lomas, which often consist of monospecific *Tillandsia* stands at locations where often no other plants are able to survive. *Tillandsia* belongs to the Bromeliaceae family and includes many highly specialized species often well‐adapted to drought and high temperature (Givnish et al., 2014; Males & Griffiths, 2017). In particular, *Tillandsia* consists of hundreds of species of so‐called grey tillandsias, which are confined to a functional ecological type called crassulacean acid metabolism (CAM) atmospheric epiphytes (Males & Griffiths, 2017). Most grey *Tillandsia* are epiphytes, and roots do not function as organ for water and nutrient uptake, but they serve as structure to fixate with the substrate. Consequently, they harvest water with highly specialized trichomes on leaf and shoot surfaces from rain or fog (Haslam et al., 2003). This adaptation for water‐uptake efficiency under extreme conditions and its combination with CAM photosynthesis is representing an efficient way to maximize water‐use efficiency. However, few grey and rootless *Tillandsia* species were able to colonize terrestrial habitats (Hesse, 2012; Koch et al., 2019; Pinto et al., 2006), and most of them are found in Chile and Peru where they grow on bare sand or gravel with the only water source coming from night‐time fog (Zizka et al., 2009).

The most abundant loma‐forming species in Chile is *Tillandsia landbeckii*, which builds up regular linear structures arranged orthogonally to the sloped desert landscape. Since the species lacks a root system, it depends on these sloped dunes (coppice dunes) to stabilize plant growth. In principle, plant growth is a balanced situation of unidirectional growth toward incoming fog facing downhill, trapping sand to stabilize growing shoots, and dieback and oversanding of the older *Tillandsia* plants. This results in dune growth and migration in an unusual upwind direction, and to optimize fog harvesting and minimize self‐competition (“fog shadow”), the vegetation is structured in rows with defined space between them depending on slope. With increasing slope, the distance between rows is decreasing; however, there is an optimal slope ranging from between 5.5 and 12.5° because sand has to be trapped to stabilize growth (Wolf et al., 2016). Such linear or banded formation patterns in arid climates have already been described, but we lack a deeper understanding of growth processes (Aponte & Flores, 2013; Borthagaray et al., 2010; Deblauwe et al., 2012; Hesse, 2012, 2014; Tlidi et al., 2018), and to our knowledge, substantial biologically relevant data are not available (Koch et al., 2019).

Based on C14 dating of buried carbon bands of subfossil *Tillandsia,* this vegetation may have persisted for thousands of years at particular sites, likely subjected to some climatic fluctuations (Jaeschke et al., 2019; Latorre et al., 2011). Substantial genetic variation between and within *T. landbeckii* populations in Chile has been demonstrated, which may have been essential for their survival in a hyperarid environment for thousands of years (Koch et al., 2019; Merklinger et al., 2020). Genetic diversity in *T. landbeckii* can be maintained by different strategies because the species easily clonally propagates with shoot offsets, but also geneflow via pollen and subsequent seed dispersal maintains and continuously creates genetic diversity (Merklinger et al., 2020). Although the formation of regular linear banding patterns may favor clonal population growth at least along those lines, given the extent of the extreme habitat over such a large distribution range, continuous local adaptation depending on sexual reproduction may be necessary. At the local scale, sexual reproduction and clonal dispersal appear to form a balanced system depending on suitable space for establishment (Koch et al., 2019) and the need to adapt to differing microniches and to survive catastrophic events wiping out local subpopulations (e.g., oversanding and long‐term absence of fog). Over the past decades, in particular, *Tillandsia* lomas dieback has been high (Schulz et al., 2010), and it has been hypothesized that this may relate to changes in fog dynamics caused by decadal natural cycles in the Pacific basin (Muñoz et al., 2016; Schulz et al., 2011), enhanced by global climate change and its effect on Chilean coastal climate in general and regional‐wide El Niño Southern Oscillation in particular (Cai et al., 2014; Del Rio et al., 2018).

We hypothesized that the combination of: (a) An extreme environment driving life at its dry limits, (b) a long evolutionary history, and (c) long‐term population persistence have developed fine‐scale adapted populations showing balanced systems integrating biotic and abiotic factors. We studied landscape genetics and elaborated on the association with fitness parameters, such as growth rate, biomass production, and flowering.

## 2 MATERIAL AND METHODS

### 2.1 Plant material, study sites, and sample plots

**FIGURE 1:**
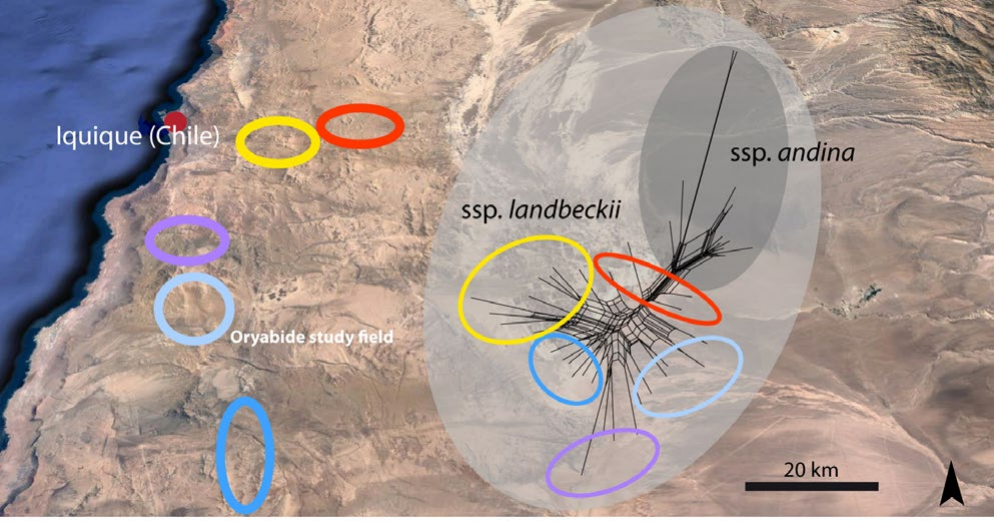
Distribution map of investigated populations of *Tillandsia landbeckii* in northern Chile. The Oyarbide study area is indicated in light blue. The corresponding SplitsTree graph based on genetic distances (AFLP dataset 1) is shown and also includes *T. landbeckii* ssp. *andina* from Peru as an outgroup

A detailed distribution survey of *T. landbeckii* lomas in northern Chile was first documented by Rundel et al. (1997). This information was used to sample a first screening set of samples near the town Iquique (Chile) along a ~80‐km‐long transect. In the center of this transect, our main study field (Oyarbide field) was located (Figure 1), which is resembling a typical study site with a well‐developed vegetation. We sampled 30 individuals from five population systems complemented with 11 individuals of *T. landbeckii* ssp. *andina* from Peru collected from 2,000 to 3,000 m a. s. l. and cultivated at Heidelberg Botanical Gardens (collection codes HEID113095, 112451a, 113094, 113099a, 113099, and 100442). The *T. landbeckii* ssp. *andina* was included as an outgroup for genetic analysis, but also as reference for growth performance comparison, because this species grows under less arid conditions with higher water (fog) availability. The plant material served as a basis for AFLP dataset 1 (amplified fragment length polymorphism) and should also demonstrate that the selected Oyarbide study field fits well within the regional patterns of genetic variation.

**FIGURE 2:**
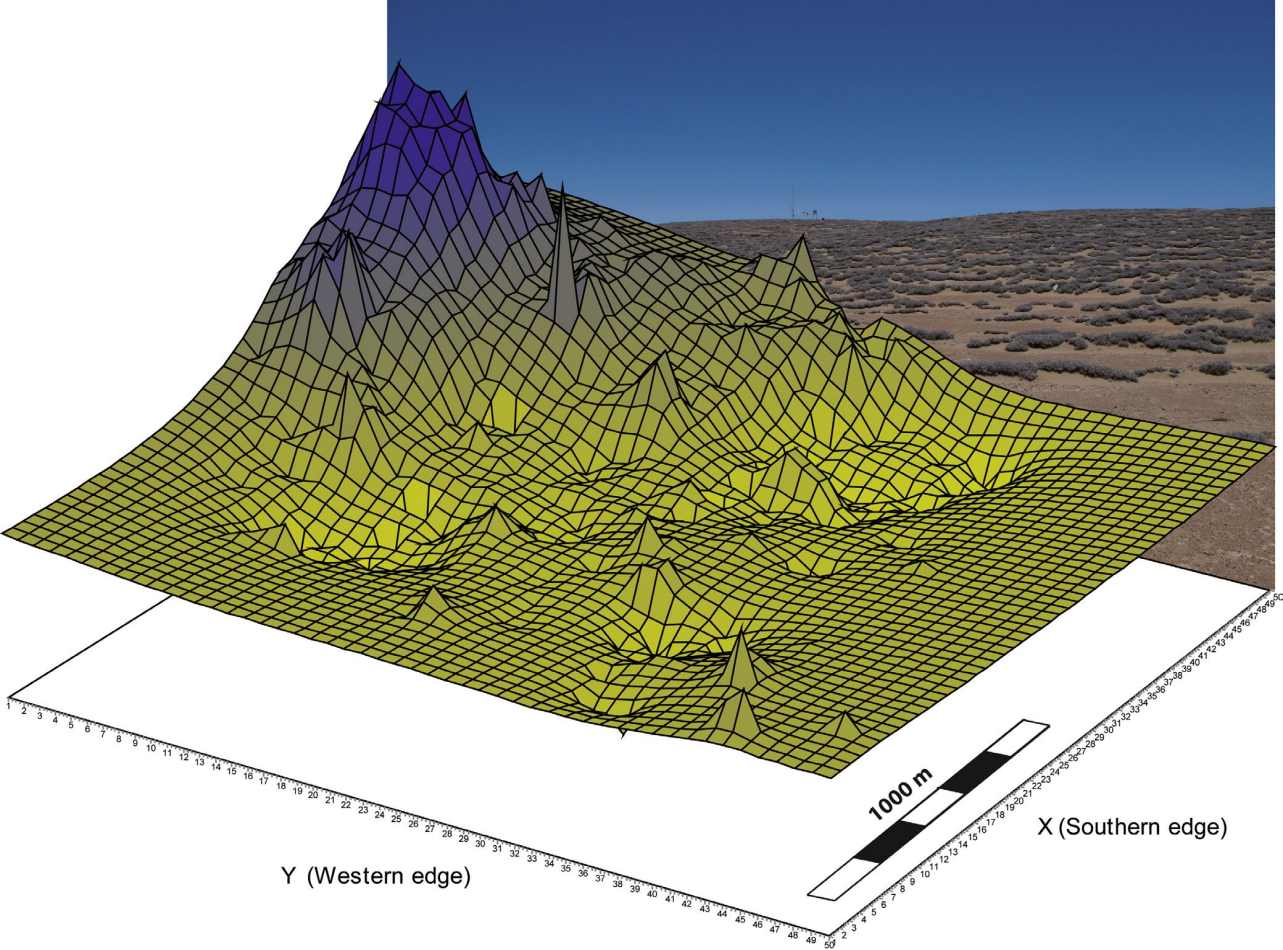
Genetic landscape of *Tillandsia landbeckii* for the Oyarbide study area. The background image is a view toward the northeast (increasing altitude and genetic diversity)

The second AFLP dataset originated from a dense sampling in a 100 × 100 m grid at the Oyarbide study field. This dataset was reported previously to study geneflow and small‐scale genetic differentiation along linearly arranged vegetation (Koch et al., 2019). This AFLP dataset 2 comprised 199 individuals of *T. landbeckii* and covered an area of ~8 km^2^. The study terrain is sloped in a southwest–northeast direction [5–12 (–30)°] (Pacific Ocean in west/southwest direction) and spans an elevational range from 1,140 to 1,212 m a. s. l. The Oyarbide study field and its *Tillandsia* vegetation consist of one vascular plant species, *T. landbeckii*. Vegetation cover was explored via high‐resolution drone‐based air photographs (see Section 2.4). Accordingly, 10 sample plots were installed over the entire field representing all five defined vegetation cover classes distributed over the entire studied population area (Figure 2). At each of these 10 sample plots, the seasonal growth of 50 plants was monitored (see Section 2.5).

For growth analysis at the Botanical Garden Heidelberg, we used six healthy individuals of *T. landbeckii* spp. *andina* (see accession codes above) and 10 individuals of *T. landbeckii* ssp. *landbeckii* from Chile (HEID 100441b, 112534, 112451b, 113098, 113097b, 113096, 100441a, 113097a, 113096b, and 100442a).

### 2.2 Molecular analysis (AFLPs)

AFLP profiles were generated for dataset 1 with 41 individuals in total (Meudt & Clarke, 2007; Vos et al., 1995). This sampling also included seven randomly selected individuals from the Oyarbide study field. Leaf material was taken from living plants in the field or at the Botanical Garden Heidelberg and immediately stored in silica gel for fast drying. Leaf material was homogenized by grinding with a pestle in liquid nitrogen. The Invisorb Spin Plant Mini kit was used for DNA extraction following the manufacturer's instructions (Invitek). The following modifications were applied to the protocol: DNA pellets were washed twice with 70% ethanol and then dissolved in 100 μl TE buffer (10 mM Tris‐HCl, 1 mM EDTA, pH 7.5) supplemented with 2 units of RNase A; RNA digestion was performed at 37°C for 1 hr. DNA quality was checked on 1% agarose gels, and concentration was assessed via fluorescence spectroscopy using a high‐sensitivity, double‐stranded DNA specific dye with the Qubit^®^ dsDNA HS Assay (Thermo Fisher Scientific). Digestion of diluted genomic DNA and ligation of dsDNA adaptors was performed simultaneously by endonucleases *Eco*RI HF and *Mse*I and T4 DNA Ligase (New England Biolabs GmbH).

PCR reactions were carried out with AmpliTaq DNA Polymerase in AmpliTag buffer II for PCR step 1 and with AmpliTaq Gold and AmpliTaq Gold Buffer (all Life Technologies) for PCR step 2 (selective PCR). Oligonucleotides and fluorescent‐labeled oligonucleotides (with fluorophores FAM, Hex, and Atto550) were obtained from biomers.net GmbH (Ulm, Germany). For this initial screening of genetic variability, a set of three combinations of selective primers was selected: (A) *Eco*RI + ACA(FAM)/*Mse*I + CAT, (B) *Eco*RI + AAC(Hex)/*Mse*I + CAT, and (C) *Eco*RI + AGC(Atto550)/*Mse*I + CAT). Prior to fragment detection, amplicons were poolplexed and purified by ultrafiltration (NucleoFast 96 PCR plate ultrafiltration kit, Macherey‐Nagel). Custom fragment detection was performed by GATC Biotech AG (Konstanz, Germany). Size calling and manual genotype calling were performed with GeneMarker 1.95 (SoftGenetics LLC). Scored data were exported as a binary data table for further analysis.

To assess the genotyping error, we included replicate samples from duplicated DNA extractions in our setup. In total, we generated 10 replicate genotypes for 10 individuals plus several negative (water) controls. Samples were assigned randomly to an experimental block (96‐well reaction plate). Sampling and design of AFLP dataset 2 (199 individuals) were based on AFLP dataset 1 and were reported previously (Koch et al., 2019). This dataset consists of three additional selective primer combinations: (D) *Eco*RI + ACA(FAM)/*Mse*I + CTC, (E) *Eco*RI + AAC(Hex)/*Mse*I + CTC, and (F) *Eco*RI + AGC (Atto550)/*Mse*I + CAT) and included 199 individuals. Therefore, we analyzed both datasets separately. Data were deposited with DRYAD (https://doi.org/10.5061/dryad.8sf7m0cjm).

### 2.3 Genetic data analysis

The AFLP analysis of dataset 1 including 41 individuals resulted in 165 loci after semi‐automatically scoring and rigorous quality control (Koch et al., 2017). The final matrix consisted of polymorphic loci only, and the error rate, according to Bonin et al. (2004), was 3.9%. To visualize the general genetic structure among populations, a parsimonious SplitsTree analysis was performed with SplitsTree vers. 4 (Huson & Bryant, 2006) using uncorrected *p*‐distances and the Jaccard index under the NeighborNet option. Bootstrap analysis was performed with 1,000 replicates.

AFLP dataset 2 consisted of 131 loci from 199 individuals (Koch et al., 2019). All individuals were georeferenced according to high‐resolution aerial photographs (Koch et al., 2019), and an accuracy of ~0.3 m was achieved. Accordingly, using an area model with detailed data on elevation at a minimum resolution of 30 cm (Wolf et al., 2016), positioning of individuals on a 100 × 100 m grid base is very precise. Genetic data of this sampling were previously reported (Koch et al., 2019), and herein, we discuss these data in the context of growth parameters. We further explored the data spatially and generated a genetic diversity landscape using Alleles in Space (AIS) version 1.0 (Miller, 2005) using the analysis “Interpolate Genetic Landscape Shape.” Geographic X‐Range and Y‐Range were separated into 50 bins each, and distance weight value was set to 1 (default parameter). The procedure was initiated by constructing a connectivity network of sample sides and assigning calculated interindividual genetic distances to landscape coordinates at midpoints of the connectivity network edges. AIS performs joint analyses of interindividual spatial and genetic information that can be applied at almost any spatial scale (Miller, 2005). From genetic landscape data, we extracted the 3‐dimensional coordinates and correlated Z‐dimension (genetic diversity) with respective elevation data, which were extracted from the high‐resolution 3D‐georeferenced landscape model. A correlation test was performed using Spearman's rank correlation coefficient, Spearman rho, and Kendall tau using R (R Core Team, 2018). Accordingly, genetic landscape data were also correlated with vegetation cover data (see Section 2.4), providing vegetation cover data on a 30 × 30 m grid resolution. Mean values of the dataset were compared using the Mann–Whitney U test in R vers. 3.5.2 because data did not follow a normal distribution.

### 2.4 Image‐based vegetation cover analysis

The vegetation cover fraction (VCF) is based on an orthomosaic from 2015 with a spatial resolution of 2 × 2 cm and three spectral bands (blue, green, and red). VCF calculation is shown in Figure 2. The orthomosaic was created by means of the structure from motion (SfM) technique (Micheletti et al., 2015; Westoby et al., 2012) with the software Agisoft PhotoScan Pro (Agisoft LLC), using several hundred pictures taken by the paraglider drone SUSI‐62 (GeoTechnic) and a Nikon D300 camera. The *Tillandsia* plants were then detected via an object‐based classification (Blaschke, 2010) with the software Trimble eCognition Developer (Trimble). First, three additional image layers were created, hereafter referred to as EDGE [extracted from the red band with the Canny edge detector (Canny, 1986) and a Gaussian convolution filter], VI (Vegetation Index, calculated by red–blue), and SI (Shadow Index, calculated by 2× red–blue). Second, a multilevel multithreshold segmentation was applied to extract objects representing the *Tillandsia* plants based on the set thresholds: minimum VI, minimum red, minimum SI, mean green, mean EDGE, mean VI, and mean blue as well as a minimum mapping unit of 35 pixels. Third, the resulting objects were further refined by merging, growing (using the thresholds SI, VI, red, and relative area of *Tillandsia* pixels), and filling small holes. Following *Tillandsia* detection, the VCF was calculated based on a 30 m × 30 m grid that represented the pixels of the digital elevation model SRTM, enabling further analyses. The VCF describes the proportion of one tile covered by *Tillandsia* plants. Our digital elevation model was also used to extract altitude data to be correlated with the genetic landscape model.

### 2.5 Growth measurements in the field and greenhouse

From each of 10 selected plots at the Oyarbide study field, 50 individuals were scored for shoot growth. Shoots were marked individually with aluminum labels and a resistant mark against sand erosion, wind, and light intensity. To correctly measure shoot growth (and not leaves), we used short bands of thread that were tied around the shoot and fixed with a drop of rubber glue. Direct inspection of the apical meristem in grey *Tillandsia* was not possible because it was always surrounded by expanding leaves. Therefore, the fixed position of the threat indicated the latest node, and the distance to the shoot tip was measured as the base line (15 October 2017). The shoot tip was defined as the most basal position of terminally diverging leaves. Then, we measured increasing distances over a period of 409 days (15 March 2018, 14 May 2018, 3 August 2018, and 28 November 2018). Measurements were performed with a high precision digital micrometer, and values were taken in mm. All sample plots were also monitored for flowering frequency in November 2018. Flowering was categorized into five classes: E 0–4, D 5–8, C 9–12, B 13–16, or A > 16 flowering shoots per 100 cm^2^.

Similarly, we analyzed the growth of *T. landbeckii* (*n* = 10) and *T. landbeckii* ssp. *andina* (*n* = 6) with plants cultivated at the Heidelberg Botanical Gardens under standard growing conditions (22 May 2017 until 23 April 2018, every 2–3 weeks; greenhouse conditions with day‐time temperature varying between 20–30°C, night‐time temperature not below 15°C, extralight to ensure a minimum of 12‐hr light exposure, daily simulation of fog water events spraying the plants in the afternoon or early morning).

### 2.6 Estimation of fresh and dry biomass

To calculate realistic biomass values for the entire study field, we investigated the correlation of volume, fresh biomass, and dry biomass. These data should be used in future to develop a model for calculating biomass directly from drone‐based 3D imaging of various *Tillandsia* fields. Photographs were taken by a drone, and the Agisoft PhotoScan software was used to process digital images and generates 3D data. The process included the following steps and settings to achieve a maximum resolution of plant surfaces: “align photos” with settings “high quality” and “generic”; “Generate dense cloud” with settings “high quality,” “mild depth filtering” and “no reuse depth filtering maps”; “Build mesh” with setting “surface type to arbitrary high,” “source data to dense cloud,” “face count to high,” and “interpolation enabled”; “Close holes” was set to 100%. A reference length was included with the images to calculate the absolute volume of subsequently harvested plant material. In total, 12 samples of varying volume were collected. All material was kept at moderate humidity (65%) for 24 hr, and fresh weight was measured accordingly. Subsequently, the plant material was dried out completely at 75°C in a drying cabinet with 4‐fold constant air removal, and dry weight was measured.

## 3 RESULTS

### 3.1 Genetic structure

AFLP dataset 1, which was analyzed with SplitsTree, provided congruent results irrespective of the genetic distance measure used. The splits graph based on uncorrected *p*‐distances is shown in Figure 1. Outgroup accessions of *T. landbeckii* ssp. *andina* from Peru were significantly separated. The relevant bootstrap value was 98% (*p*‐distance) or 93% (Jaccard distance). Furthermore, there was substantial regional clustering of individuals according to the five selected (meta)populations, and AFLP data also indicated some closer relationship of the two northern populations (La Islas, red; Guan, yellow), and the three southern populations (Guanako, violet; Oyarbide, light blue; Pajares, blue). Individuals from the Oyarbide study field (Figure 1, light blue) clustered together without any obvious outlier.

**FIGURE 3:**
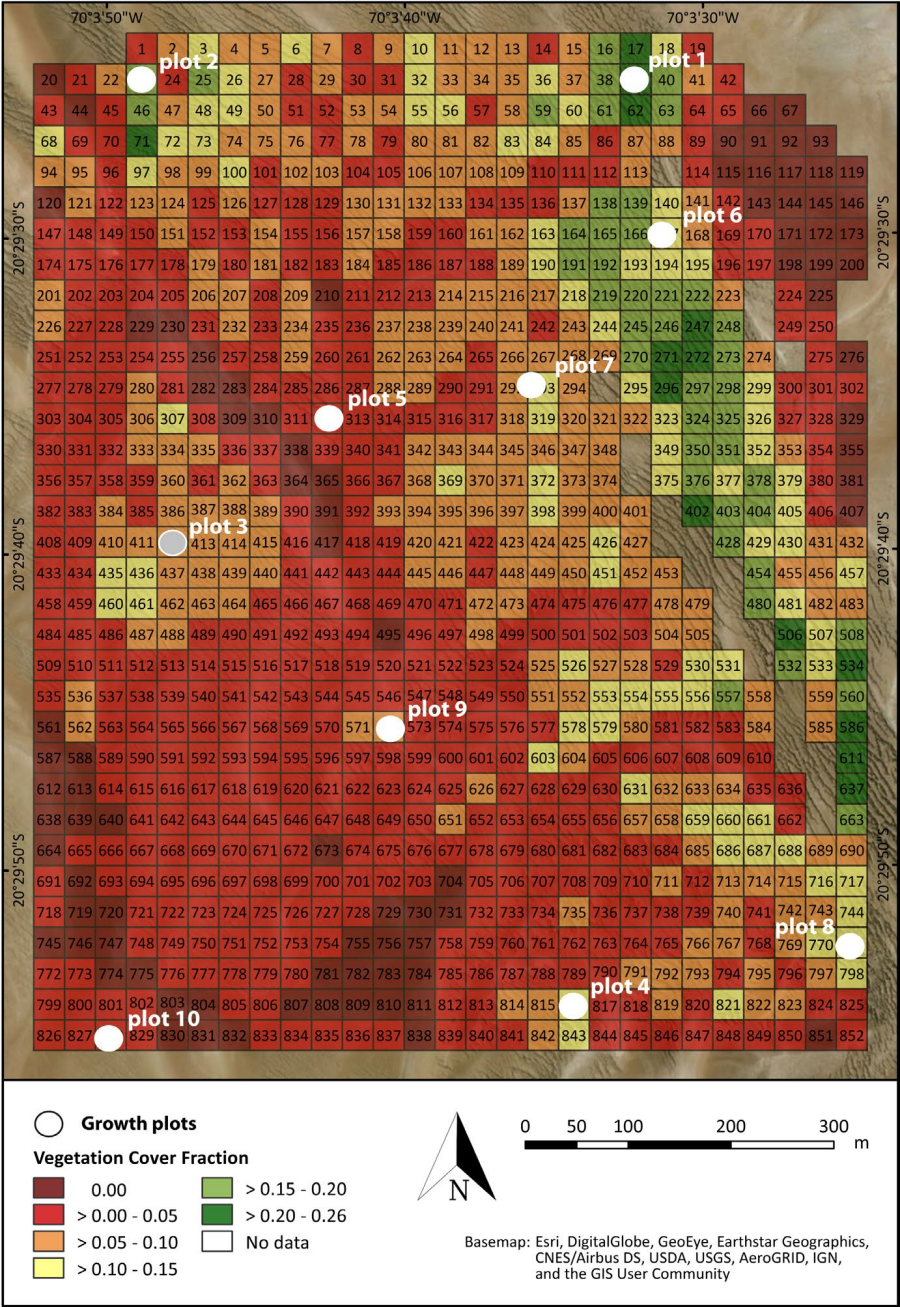
Estimation of vegetation cover fraction (VCF) of *Tillandsia landbeckii* at Oyarbide. Processing of the aerial orthomosaics aggregated data on a 30 × 30 m grid scale. The location of the 10 selected study plots is indicated. Data from plot 3 were excluded

For AFLP dataset 2, the genotyping error rate calculated according to Bonin et al. (2004) was <0.5% (Koch et al., 2019). Calculation of the genetic landscape using AIS resulted in a 3D graph as shown in Figure 3. This graph clearly shows decreasing genetic diversity toward the southwest facing the Pacific Ocean along a gradient of ~2 km and an elevational cline of 128 m. This pattern followed the sloped terrain. The correlation analysis of genetic diversity and elevation (*n* = 644 data points extrapolated based on the 50‐bit grid from AIS, Figure 2) resulted in a weak (*r* = .2275/.1642; Spearman rho/Kendall tau, respectively) but significant (*p* = .014/.012) correlation (Table 1). Correlation between genetic diversity and vegetation cover fraction was not significant (Table 1). Both AFLP datasets including coordinates were uploaded to DRYAD (https://doi.org/10.5061/dryad.8sf7m0cjm).

**TABLE 1:** Pairwise correlation of parameters from the entire Oyarbide study field (100 × 100 m grid size): genetic diversity, elevation, and vegetation cover fraction (VCF)

Pair	Spearman rho	Kendall tau
Genetic diversity/elevation	**0.227 (.014)**	**0.164 (.012)**
Genetic diversity/VCF	0.106 (.358)	0.077 (.331)
Elevation/VCF	**0.537 (.001)**	**0.398 (.001)**

Significant correlations are indicated in bold.

*p‐*Values are shown in parentheses.

### 3.2 Vegetation cover

Values of total vegetation cover within a given part of 30 × 30 m ranged from 0% to 26%. This is comparable with values reported by Rundel et al. (1997) ranging from 5% to 31% (with one outlier at 37%). Vegetation cover was grouped into six categories in 5% vegetation cover intervals. The map is shown in Figure 3 in which a gradient toward the sloped terrain in the southwest direction facing the Pacific Ocean is evident. The selected plots (Figure 3) represent all five vegetation cover categories with growing *Tillandsia*. For the entire Oyarbide study field, there was a moderate (*r* = .5373/.3984; Spearman rho/Kendall tau, respectively) and highly significant (*p* < .001) correlation between VCF and elevation (Table 1). Respective graphs and scatterplots are shown with the appendix (Figures [Link], [Link], [Link]).

### 3.3 Growth and biomass

**FIGURE 4:**
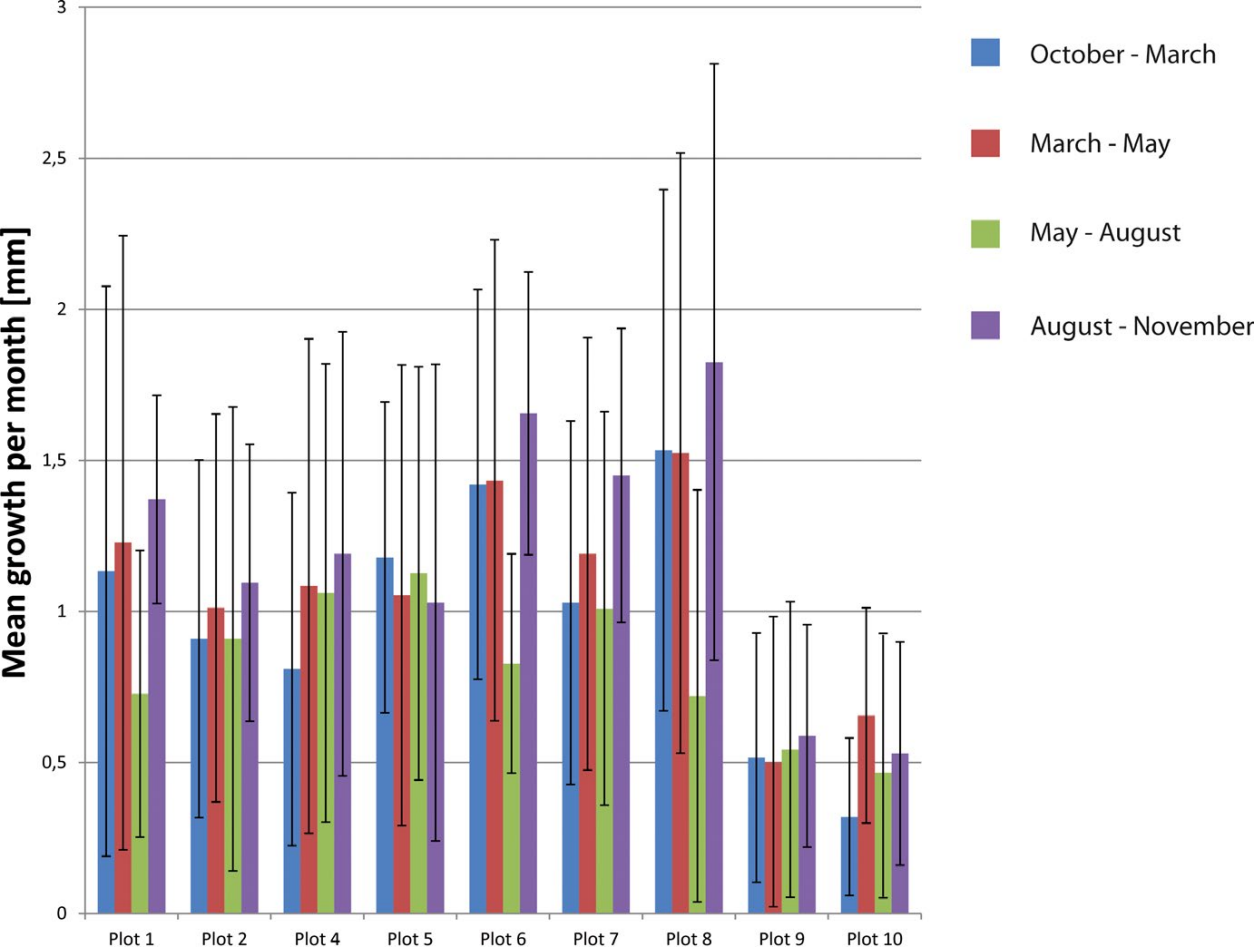
Growth of *Tillandsia landbeckii* from the nine study plots calculated per month and for the four monitoring periods separately. Standard deviation is indicated. Total number of individuals analyzed is shown in Figure 5

**FIGURE 5:**
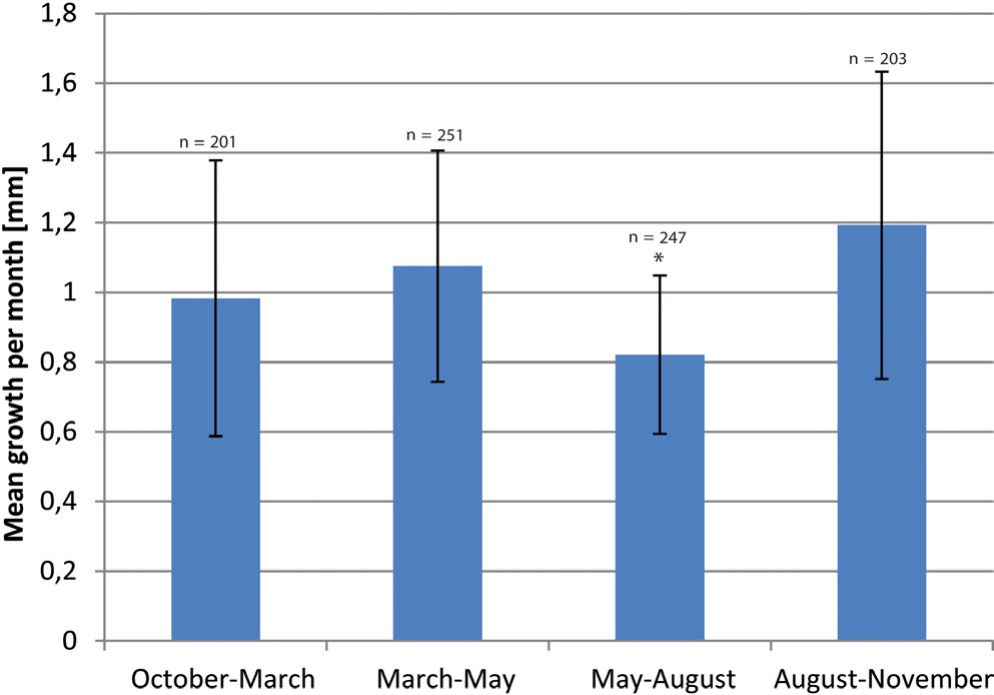
Mean growth per month aggregated for any data point for the entire Oyarbide study field and nine permanent plots and four quarterly periods (Q1: October–March, Q2: March–May: Q3: May–August; Q4: August–November. The only significant shift in mean growth was from Q3 to Q4 (Mann–Whitney *U* test: *p* = .05). Growth values of plots among seasons were highly correlated comparing Q1, Q2, and Q4 (*p* < .01, Kendall tau) but not with Q3 (not significant) (Table 2)

**FIGURE 6:**
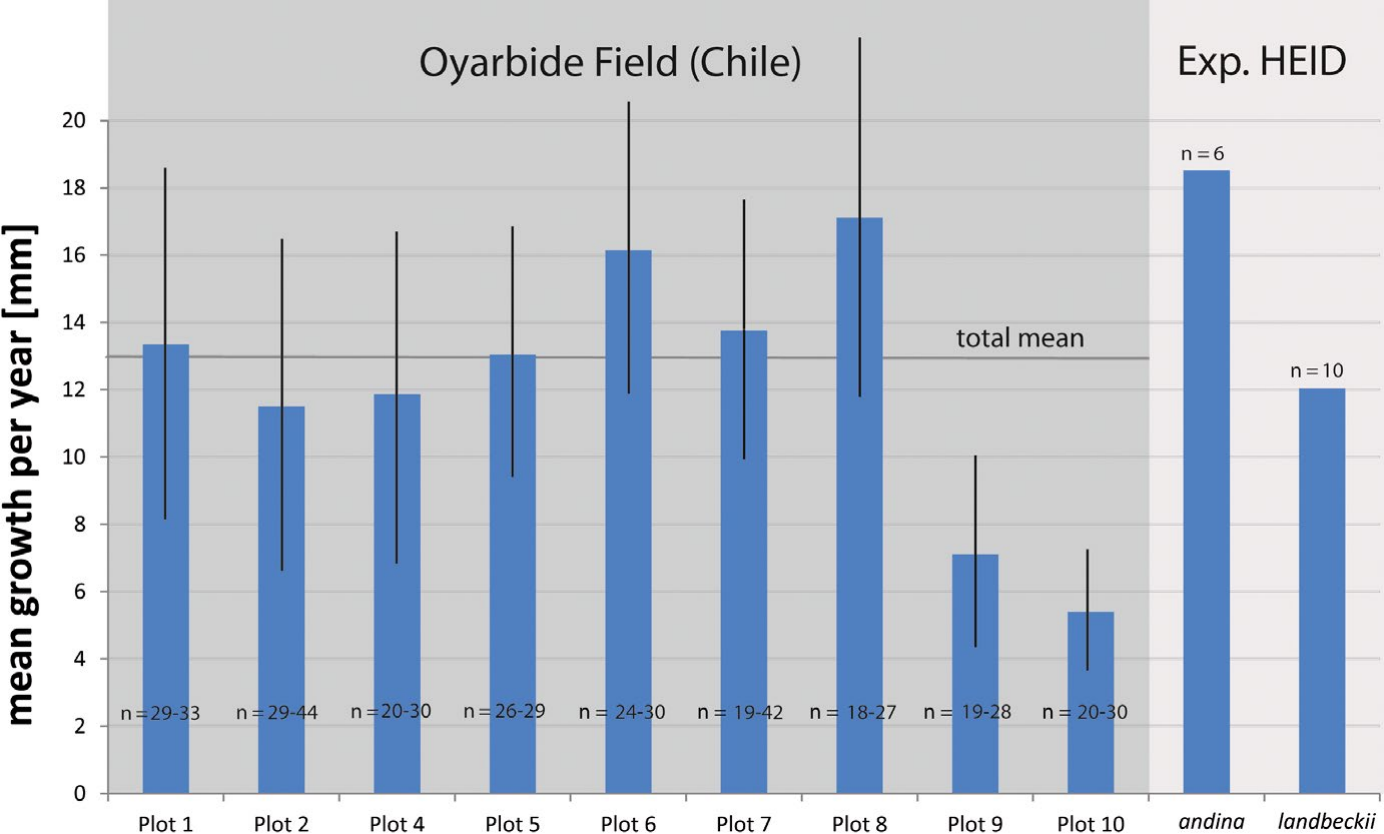
Total growth per year aggregated for the nine study plots at Oyarbide. Standard deviation, range of number of individuals analyzed, and total mean of growth per year are indicated. The mean values of yearly growth of *Tillandsia landbeckii* and *T. landbeckii* ssp. *andina* from the growing experiment at Heidelberg Botanical Garden (HEID) are also shown

From the 10 study plots at Oyarbide, plot 3 was destroyed during the first quarter, and we were not able to reliably restore the data. Consequently, growth data from nine plots are shown and discussed. For these remaining plots, various marks failed during the single scoring period (mostly due to strong winds) and were newly fixed and calibrated. Consequently, the number of data points varied when comparing among plots and time points for scoring. Yearly growth data (per plot and total) were calculated based on the mean values from each monitoring season, and thereby, including any data point, rather than using only those plants without any missing data over the whole scoring period. Data were deposited with DRYAD (https://doi.org/10.5061/dryad.8sf7m0cjm). Raw data of mean growth per month [mm] are shown in Figure 4 for all plots, and data were analyzed and compared according to the four time periods. There was a significant signal indicating that growth was slower in May–August (*p* = .05 for plots 1, 6, and 8; Mann–Whitney *U* test) compared with the other three time periods. This weak signal was retained when data were aggregated according to time period (Figure 5), with mean growth rate from May to August being significantly lower than that in the following period from August to November with maximum growth (*p* = .05, Mann–Whitney *U* test, all other pairwise comparisons do not show a significant difference; Table 2). This may indicate a growth response to higher fog occurrence and respective water supply with a peak season between July and September (Del Rio et al., 2018; long‐term data from climate stations in the same area but not from Oyarbide). More precisely in terms of geography (but not long‐term data), however, fog collection data from 1 year at Oyarbide at four sites at different altitudes revealed a peak fog season between August and November (Zanetta et al., 2015), which is consistent with our growth measurements, indicating minimum growth preceding peak fog season and then followed by maximum growth observed from August to November (Figure 5). All individual growth data were aggregated, and mean growth per year was calculated for the various plots (Figure 6). The total mean of yearly growth over the entire Oyarbide study field (*n* = 293 individuals) was 13 mm per year (Figure 6). This corresponded well with the mean growth per year of *T. landbeckii* cultivated at Heidelberg Botanical Garden (12 mm per year, *SD* = 6.9 mm, *n* = 10). As expected, *T. landbeckii* ssp. *andina* from high elevations in Peru showed an even higher mean growth rate of 18.5 mm per year (*SD* = 17 mm, *n* = 6).

**TABLE 2:** Pairwise correlation of growth for the quarterly growing periods (Q1–Q4) considering all individuals studied at Oyarbide reference plots

Pair	Spearman rho	Kendall tau
Q1:Q2	**0.850 (.006)**	**0.722 (.006)**
Q1:Q3	0.283 (.463)	0.167 (.612)
Q1:Q4	**0.800 (.014)**	**0.667 (.013)**
Q2:Q3	0.117 (.677)	0.001 (.990)
Q2:Q4	**0.950 (.001)**	**0.833 (.009)**
Q3:Q4	0.183 (.644)	0.056 (.919)

Significant correlations are indicated in bold.

*p‐*Values are shown in parentheses.

**FIGURE 7:**
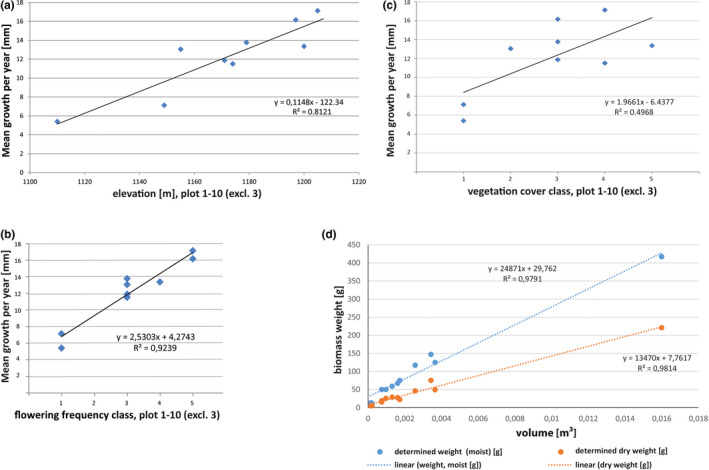
Scatterplots of correlation analyses comparing yearly growth, altitude, vegetation cover fraction, and flowering density at the nine study plots at Oyarbide. (a) Yearly mean growth with altitude, (b) yearly growth with increasing flowering density (categories a–e), and (c) yearly growth with increasing vegetation coverage. Refer to Table 3 for significance and correlation values. (d) Linear regression of fresh and dry biomass determination of *Tillandsia landbeckii* for defined sample volumes

Yearly growth rate in the various plots showed a strong correlation with elevation and flowering frequency (Figure 7a,b), indicating that plant growth and flowering increase with increasing elevation at the Oyarbide study field (Figure 3). The correlation of growth per year with total vegetation cover was less pronounced and not significant, but it followed a similar trend as expected (Figure 7c). Respective *p*‐values are provided in Table 3. The selected plots also showed exclusively significant correlations among the parameters elevation, vegetation cover, and flowering (*p* = .05 or .01) (Table 2). Respective graphs and scatterplots are shown in the Appendix S1.

**TABLE 3:** Pairwise correlation of parameters from the nine study plots at Oyarbide

Pair	Spearman rho	Kendall tau
Growth/elevation	**0.883 (.003)**	**0.722 (.006)**
Growth/VCF	0.385 (.307)	0.278 (.321)
Growth/flowering	**0.823 (.006)**	**0.712 (.012)**
Elevation/VCF	**0.734 (.024)**	**0.588 (.036)**
Elevation/flowering	**0.936 (.001)**	**0.836 (.003)**
VCF/flowering	**0.700 (.036)**	**0.586 (.050)**

Significant correlations are indicated in bold.

*p‐*Values are shown in parentheses.

Abbreviation: VCF, vegetation cover fraction.

If we consider that seasonal growth variation demonstrates sensitivity to fog occurrence, then we may hypothesize that the clinal variation in growth along the elevational gradient is also caused by increasing water availability along the same elevational gradient. How an elevational cline may affect fog distribution and availability has not been studied experimentally, but it should be mentioned here that with increasing elevation, the incline also increases over the entire study field from c. (0) 5 to 12 (30)° (mean 9.3°), and thereby eventually decreasing a direct fog shadow caused by the plants in the front row (Borthagaray et al., 2010).

Calculation of fresh and dry biomass, respectively, from calculated volumes of plant material also showed a linear relationship (Figure 7d). The ratio of fresh:dry weight was 1.85:1. From our data, we calculated a dry weight of ~13.75 kg per m^3^ plant material. A mean vegetation height of 25 cm and total mean vegetation cover at the Oyarbide field of ~5.5% (calculated from 30 × 30 m grid‐based mean coverage, Figure 2) resulted in an average biomass of 189 g/m^2^ dry weight [1.890 kg/ha]. Applying our measured average growth of 13 mm per year to linearly arranged *Tillandsia* individuals (rows of ~1 m), which can be considered to be at least 75–100 years in age, the yearly biomass production was ~25.2–18.9 kg/ha (1.2% biomass increase per year).

## 4 DISCUSSION

### 4.1 Genetic data: from evolutionary history to local differentiation

*T. landbeckii* lomas in the Chilean Atacama Desert show varying degrees of genetic connectivity over their entire distribution range (Merklinger et al., 2020). This pattern is underpinned by three main genetic clusters with a peripheral and small southern cluster, a restricted northern cluster, and a major and geographically central cluster, together demonstrating significant correlation of genetic and geographic distance. Herein, we focused on a metapopulation system from the central genetic cluster, and we observed close genetic relationships between populations from our study region (Figure 1) and significant genetic connectivity (Koch et al., 2019). Long‐term evolutionary history of *T. landbeckii* populations in the Atacama Desert remains unknown, and observed present‐day large‐scale genetic patterns may be explained either by a formerly continuous metapopulation that became fragmented or by colonization (maybe multiple times) of outlier populations from source gene pools (Merklinger et al., 2020). It has also been demonstrated that both sexual/clonal propagation and dispersal play a role in successful plant establishment at larger geographic scales (Merklinger et al., 2020) as well as at the regional and populational levels (Koch et al., 2019).

Large‐scale genetic differentiation due to migration, colonization, and extinction may mirror the effect of Pleistocene climatic fluctuations, drastically altering extreme aridity, with the recent less arid interglacial phases 75–135 and 175–225 thousand years ago (Marine Isotope Stage 5 and Marine Isotope Stage 7) eventually opening larger corridors for migration in north–south/south–north directions (Ritter et al., 2019). Apart from this information reflecting hundreds of thousands of years, there is also evidence from stratigraphic and stable carbon isotope analysis of subfossil banding patterns inside the dunes that populations from the central and northern genetic cluster represent a vegetation type that has persisted over at least 3,000 years (Jaeschke et al., 2019; Latorre et al., 2011). Stratigraphic evidence came from distinct cap carbonates, which may have evolved by oxidation of calcium oxalate, and it was concluded that there must have been few past changes in advective fog in northern Chile that could potentially span the last 3,500 years and may have effected *T. landbeckii* lomas at the local scale.

Fine‐scale genetic data from the present study filled a gap between these ancient and evolutionary history spanning thousands and tens of thousands of years and the recent vegetation history with documentation of rapid decline of Chilean–Peruvian fog ecosystems since the mid‐1970s (Schulz et al., 2010). A systematically conducted survey focusing on a *T. landbeckii* fog oasis close to our Oyarbide study site from 1955 to 1997 calculated a total loss of living *T. landbeckii* lomas of about 35% (Osses et al., 2007; Schulz et al., 2010). The underlying causal eco‐atmospheric links are not yet fully understood (Latorre et al., 2011), but changes in the intensity of fog fluxes are likely the driving force (Latorre et al., 2011; Pinto et al., 2006). The prevailing hypothesis is that, during recent decades, the southeast Pacific anticyclone has intensified, which is linked to a greater subsidence of air, generating a shallower marine layer with a decrease in the cloud‐top altitude of 100 m decade^−1^ (Muñoz et al., 2016). The latter might cause a decrease in fog presence at the Oyarbide *Tillandsia* field and its consequent reduction in water input. In addition, these prevailing conditions in the ocean–atmosphere interactions may be intensified by the effects of global warming (Falvey & Garreaud, 2009).

**FIGURE 8:**
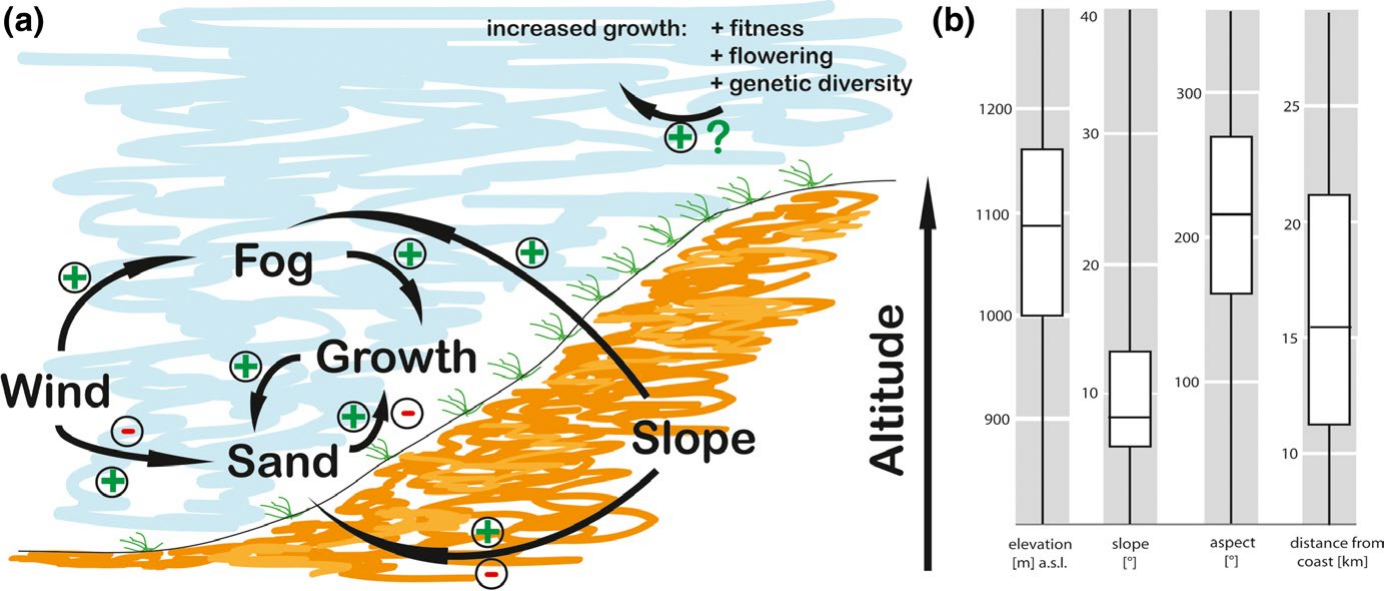
Schematic of factors potentially influencing *Tillandsia* growth and fitness. (a) The diagram indicates postulated positive and negative interactions between abiotic factors and *Tillandsia* growth. For most parameters, there is an optimal range (see b) resulting in positive and negative interactions. It is shown here that increased growth is related to increased fitness parameters such as flowering and increased genetic diversity. A positive feedback loop has not yet been observed. (b) Shows the environmental niche of *T. landbeckii* fields from the entire study area around Iquique in northern Chile (see Figure 1; redrawn boxplots diagrams from Wolf et al., 2016)

Based on our measured average growth of 13 mm per year in linearly arranged *Tillandsia* lomas, those rows are at least 75–100 years in age and therefore predate the general trend of recent decline. Consequently, we would expect our study field at Oyarbide to be characterized by the optimal geophysical parameters describing *T. landbeckii* loma distribution (Figure 8b). Indeed, mean values at Oyarbide for distance from coast, elevation, slope, and aspect (11 km, 1,174 m a.s.l., 8.72°, and 230°, respectively) fit well within the overall range of parameters documented for this vegetation. The genetic landscape at Oyarbide (Figure 2) is significantly correlated with elevation as calculated from our landscape model (Table 1, Figure S2A), and VCF was also significantly correlated with elevation (Table 1). We take this result as evidence of genetic differentiation and its spatial distribution resulting from the past 100+ years and thereby might have integrated environmental fluctuations from this period.

Additional evidence supporting this view was from the calculation of total biomass production. For the Oyarbide study field, which spanned several square kilometers, we calculated a mean total dry biomass of *T. landbeckii* of 1,890 kg/ha with a yearly production rate of ~25.2–18.9 kg/ha. Earlier rough estimates were in a similar range with 717–1,460 kg/ha for *T. landbeckii* (Rundel et al., 1997) and 1,255–1,616 kg/ha for *T. latifolia* (Masuza, 1982), a larger terrestrial species from southern Peruvian fog oases (Ogawa, 1986; Rundel & Dillon, 1998). The productivity rate of 25.2–18.9 kg/ha is low and reflects the ecological limits of the ecosystem. Planted grassland, for example, has up to 500 times higher rates (790–10,706 kg/ha/year; Henschell et al., 2015), and even in arid regions dominated by *Euphorbia tirucalli,* rates were reported at 2,200 kg/ha/year (Eshel et al., 2011) or 224–480 kg/ha/year in ungrazed desert communities of *Rhanterium epapposum* (Brown, 2002). For high‐productivity stands in arid and semi‐arid areas, Le Houérou and Hoste (1977) estimated that, for each mm of precipitation received, 4 kg of phytomass was produced per ha. For our area, this would correspond to 4–6 mm precipitation per year supplied as fog, which is lower than the general hyperarid area MAP of <25 mm (Thomas, 2011). However, there are limited studies that translated fog occurrence measured vertically by fog collectors (passive standard fog collectors, SFC; Schemenauer & Cereceda, 1994) into horizontally plant‐available precipitation equivalents (e.g., Westbeld et al., 2009). A combined modeling and approximation approach calculated a maximum of about 25 lm^−2^ per year water availability for *T. landbeckii* from fog from Cerro Guanaco (10 km north of the Oyarbide study field) with about 0.8 mm of rain per year (Westbeld et al., 2009).

Therefore, our results—considering standing biomass from past decades and also actual growth rates—might demonstrate the environmental footprint over the last 100 years or more. They also demonstrate the optimal growth of *T. landbeckii* individuals, populations, and the entire loma vegetation since growth was not different to that of plants cultivated under horticultural conditions (Figure 6). Our results also indicate that levels of on‐site water availability may be close to an equivalent of 25 mm rainfall, marking the transition from hyperarid to arid ecosystems. Therefore, we may consider *T. landbeckii* lomas to be remnants of a formerly arid vegetation, but with the ability to evolutionarily persist under hyperarid conditions due to specialized biological features (e.g., specialized trichomes and CAM metabolism) with the prerequisite of a fog system. Considering the above summarized information, the genetic landscape at Oyarbide (Figure 2) may indeed reflect genotype‐specific distribution of phenotypes as herein characterized by growth rates with a significant correlation of genetic diversity, growth rates, and occurrence at higher elevations (most likely correlated with increased amounts of fog water availability in this particular study field and perhaps the decrease in UV intensity of plants growing within the fog zone).

### 4.2 Extreme environment and small‐scale local adaptation

Using a model‐based approach, Borthagaray et al. (2010) postulated that distance between linearly arranged rows of *Tillandsia* decreases with increasing slope. Therefore, increased vegetation cover should be associated with increased density of linear structures with elevation at our study site (since slope increases toward the northeast) (Figure 2). Indeed, we were able to observe this correlation (Figure 3, appendix Figure S3). A biological explanation for this phenomenon is illustrated in Figure 8. Pattern formation is most likely controlled by fog fluxes and sand dynamics only. When slope increases, the effect of fog shadow between *Tillandsia* rows decreases, and therefore, distance between rows also decreases. Since *Tillandsia* grows toward the incoming fog parallel to the dieback of older shoots and generally does not develop roots to stabilize the shoot/leaf system, the entire system depends on sand transported by the wind. Plants then function as a sand trap, and dune formation and migration faces the direction of the wind (Latorre et al., 2011). However, this system is optimally balanced with moderate slope (Figure 8, Wolf et al., 2016).

This hypothetical growth scenario would require respective fine‐tuned growth rates. It is not only that optimal fog availability may maximize growth rate, but under these circumstances, plants must grow faster owing to the deposition of sand along moderately sloped dune systems. If plants grow slowly, then they will be covered with sand, whereas overly rapid growth will result in an unstable shoot system. We tested this hypothesis with nine study plots and found exactly the correlations expected (Figure 7a, Table 3). This growth pattern was not different if we consider the seasonal variation in growth rate (Figure 5, Figure S1).

We lacked detailed data on yearly fog deposition along transects, but a local network of seven climate stations (THIES) within a transect between ~3 km and ~15 km from the coast toward the Oyarbide study field has been employed to measure fog water quantities at a height of 2 m using standard fog collectors (SFC) since late 2019. In‐depth analysis of the data will be published in future studies when the data can be analyzed over longer time intervals. Previously reported results from SFCs in this region demonstrated fog water equivalents ranging from 2 L/m^2^ at 980 m a.s.l., 5 L/m^2^ at 1,035 m a.s.l., 9 L/m^2^ at 1,090 m a.s.l., to 13 L/m^2^ at 1,210 m a. s. l. with increasing discrepancy (Latorre et al., 2011). It has been also shown, with the same populations, that growth rates may be positively affected by fog‐mediated nutrition with phosphate (PO_4_^−3^) but without any effect of nitrogen (González et al., 2011). Here, growth rates varied between 0.01 mm/day (3.7 mm/year) and a maximum of 25.6 mm/year, which did not increase further with increasing PO_4_^−3^ input (González et al., 2011). Growth values fit very well with our estimates (Figures 4 and 6) and may indicate and confirm maximum growth rates.

Neither phosphate nor nitrogen supply is related to the distance from the coast or fog water equivalents but rather appears to be more affected by local conditions. We interpreted this as further strong evidence of fog water supply, rather than nutrient deficiency, limiting *Tillandsia* growth and fitness in its natural stands considering the herein observed fine‐scaled *T. landbeckii* growth rates and patterns also being correlated with elevation (and fog water supply). Although plants easily clonally propagate and distribute, genetic data indicate substantial small‐scaled differentiation (Figure 2) but also regional (Koch et al., 2019) and larger‐scale genetic differentiation (Merklinger et al., 2020) with signatures of significant outbreeding and geneflow. This raises questions regarding the extent to which the observed structured fitness patterns (growth and flowering; Tables 1 and 3) are explained by individual phenotypic plasticity in response to varying fog water supply or genotype‐specific morphotypes, which are mixed and dispersed in a given population. A transplantation experiment among two sites 11–30 km north of our study field demonstrated the dominant effect of the local environment on growth parameters, and similar growth patterns irrespective of origin and phenotypic variation did not change at home or away sites (González et al., 2011). This phenotypic plasticity was demonstrated at Oyarbide in all nine study plots and followed yearly fluctuations (Figures 4 and 5).

There have been few studies focusing on the role of atmospheric inputs, such as nutrient dynamics and water, on atmospheric and rootless *Tillandsia* (e.g., Abril & Bucher, 2009; González et al., 2011; Latorre et al., 2011; Richardson et al., 2000) showing that plants are able to adjust elemental stoichiometry with changing environmental conditions and by doing so respond to their local environment across such gradients of water and nutrient supply (see e.g., Geange et al., 2017). Plasticity in leaf functional traits may tend to enable *T. landbeckii* populations to shift their mean trait values not only while clonal propagules are distributed along environmental gradients but leaf trait and growth plasticity may also buffer and thereby tolerate changing environmental conditions under climate change (Henn et al., 2018). The rapid recent decline of *Tillandsia* lomas, therefore, might indicate a severe threat to loma vegetation since adaptation is not fast enough to respond and phenotypic plasticity is not sufficient to shift mean trait values. However, this question needs to be addressed in future studies.

We hypothesize for our study system of terrestrial *Tillandsia* lomas that trait plasticity, herein measured as growth, biomass production, and flowering, is an important mechanism for enabling an entire ecosystem to better tolerate changing environmental conditions under climate change. It has been concluded that the ability to adjust its phenotype to climate change is especially important in cold biomes where climate is rapidly changing (De Boeck et al., 2019), but we show that this may also be true for ecosystems growing under hyperarid/arid conditions. Observed patterns of phenotypic and genetic variation are the result of long‐term adaptation processes over thousands of years, and this setup enabled the entire ecosystem to survive and, eventually, constantly move along a north–south axis following optimal conditions (Merklinger et al., 2020). Increased and drastic local decline might reflect the severe threat from major global change toward environments, and these long‐lived plants that cannot exceed their rate of adaptation through evolutionary processes are limited by their current phenotypic plasticity.

## CONFLICT OF INTEREST

None declared.

## AUTHOR CONTRIBUTION

**Marcus A. Koch:** Conceptualization (lead); Data curation (lead); Formal analysis (equal); Funding acquisition (equal); Investigation (lead); Methodology (lead); Project administration (lead); Resources (lead); Software (equal); Supervision (lead); Validation (equal); Visualization (equal); Writing‐original draft (lead); Writing‐review & editing (lead). **Clara Stock:** Data curation (equal); Investigation (equal); Methodology (equal). **Dorothea Kleinpeter:** Data curation (equal); Investigation (equal); Methodology (equal). **Camilo del Río:** Data curation (equal); Investigation (equal); Methodology (equal); Writing‐original draft (supporting). **Pablo Osses:** Formal analysis (equal). **Alexander Siegmund:** Formal analysis (equal); Investigation (equal). **Felix F. Merklinger:** Formal analysis (equal). **Dietmar Quandt:** Investigation (equal).

## Supporting information

Figure S1A

Figure S1B

Figure S2A

Figure S2B

Figure S2C

Figure S3

Figures S1–S3

## Data Availability

Data are available from the Dryad Digital Repository: https://doi.org/10.5061/dryad.8sf7m0cjm.
