# Supplementary figures and images for: Vegetation growth and landscape genetics of Tillandsia lomas at their dry limits in the Atacama Desert show fine‐scale response to environmental parameters
Source: Ecol Evol. 2020 Oct 28;10(23):13260–74. doi: 10.1002/ece3.6924 (PMC7713976; doi:10.1002/ece3.6924)

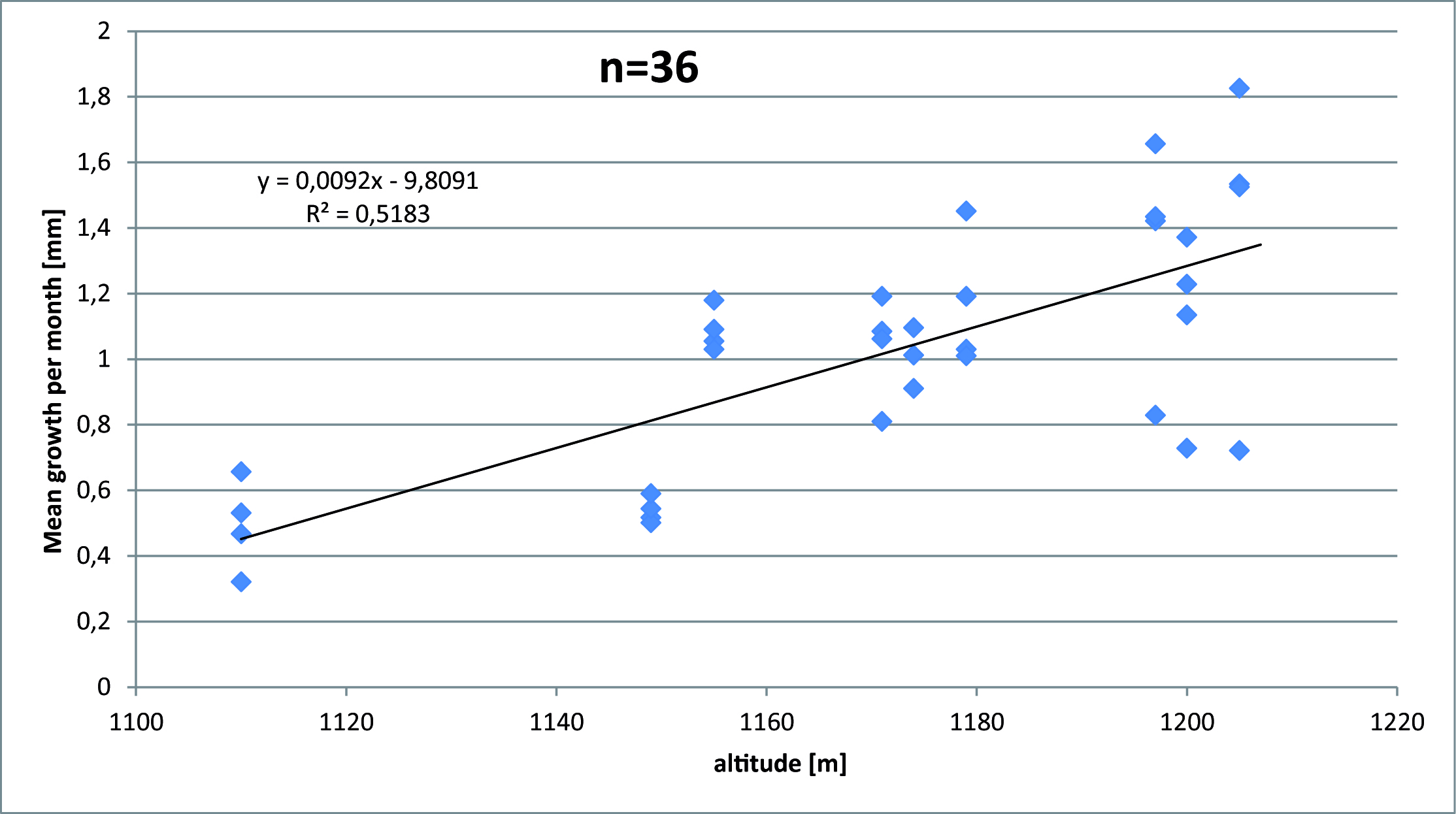

Supplement: Supplementary file 1 — Figure S1A [file ECE3-10-13260-s001.jpg]

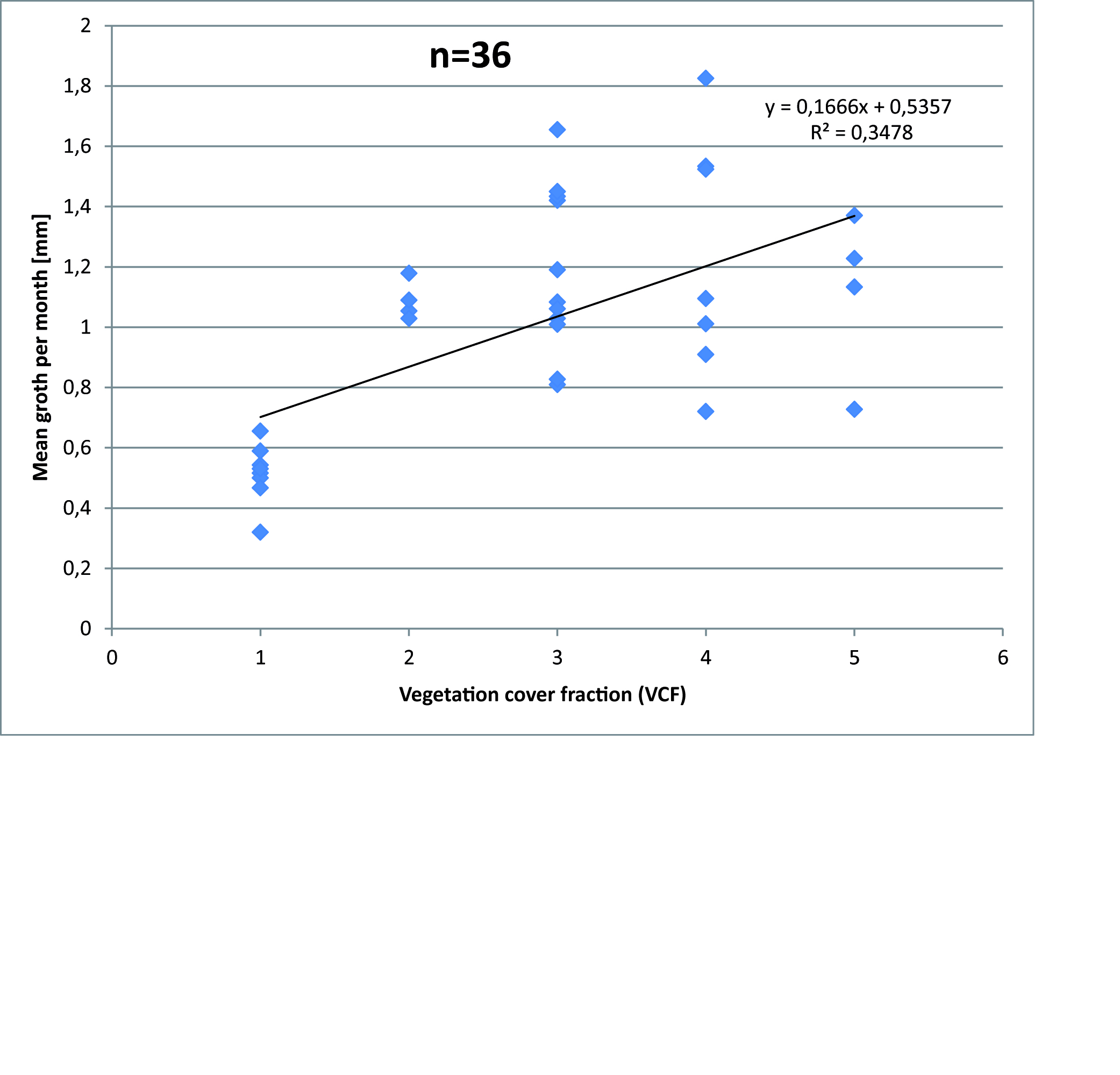

Supplement: Supplementary file 2 — Figure S1B [file ECE3-10-13260-s002.jpg]

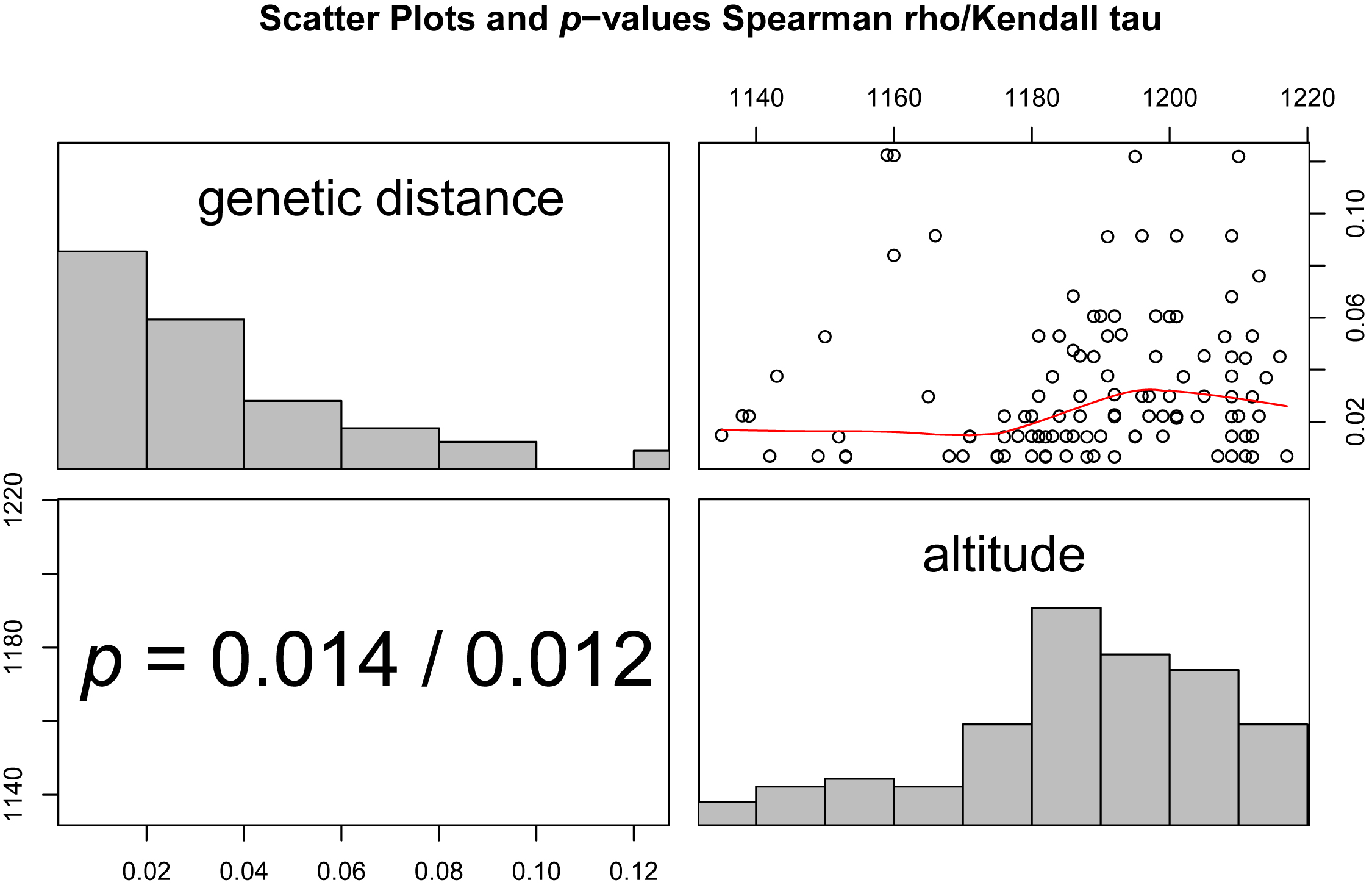

Supplement: Supplementary file 3 — Figure S2A [file ECE3-10-13260-s003.jpg]

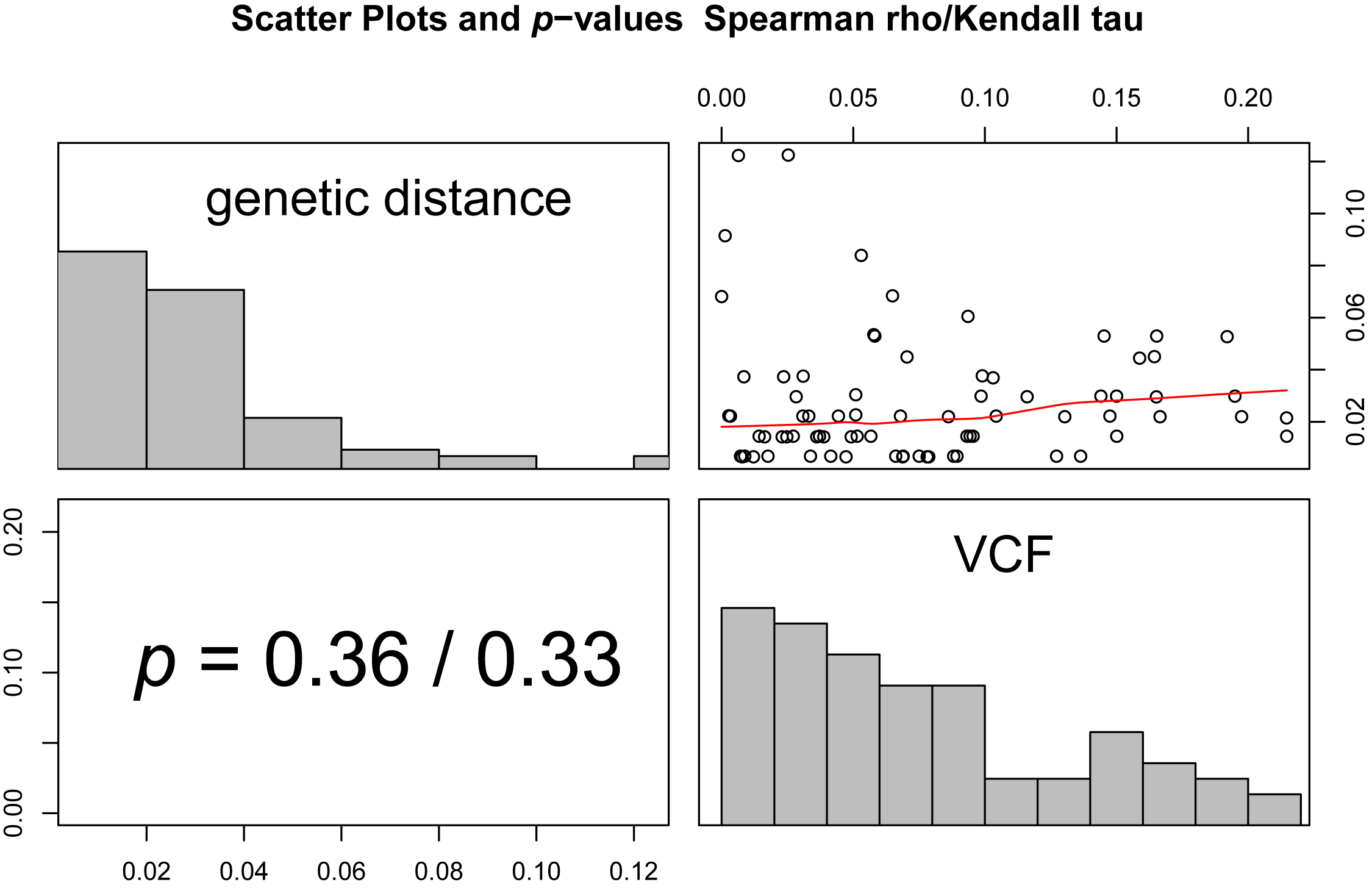

Supplement: Supplementary file 4 — Figure S2B [file ECE3-10-13260-s004.jpg]

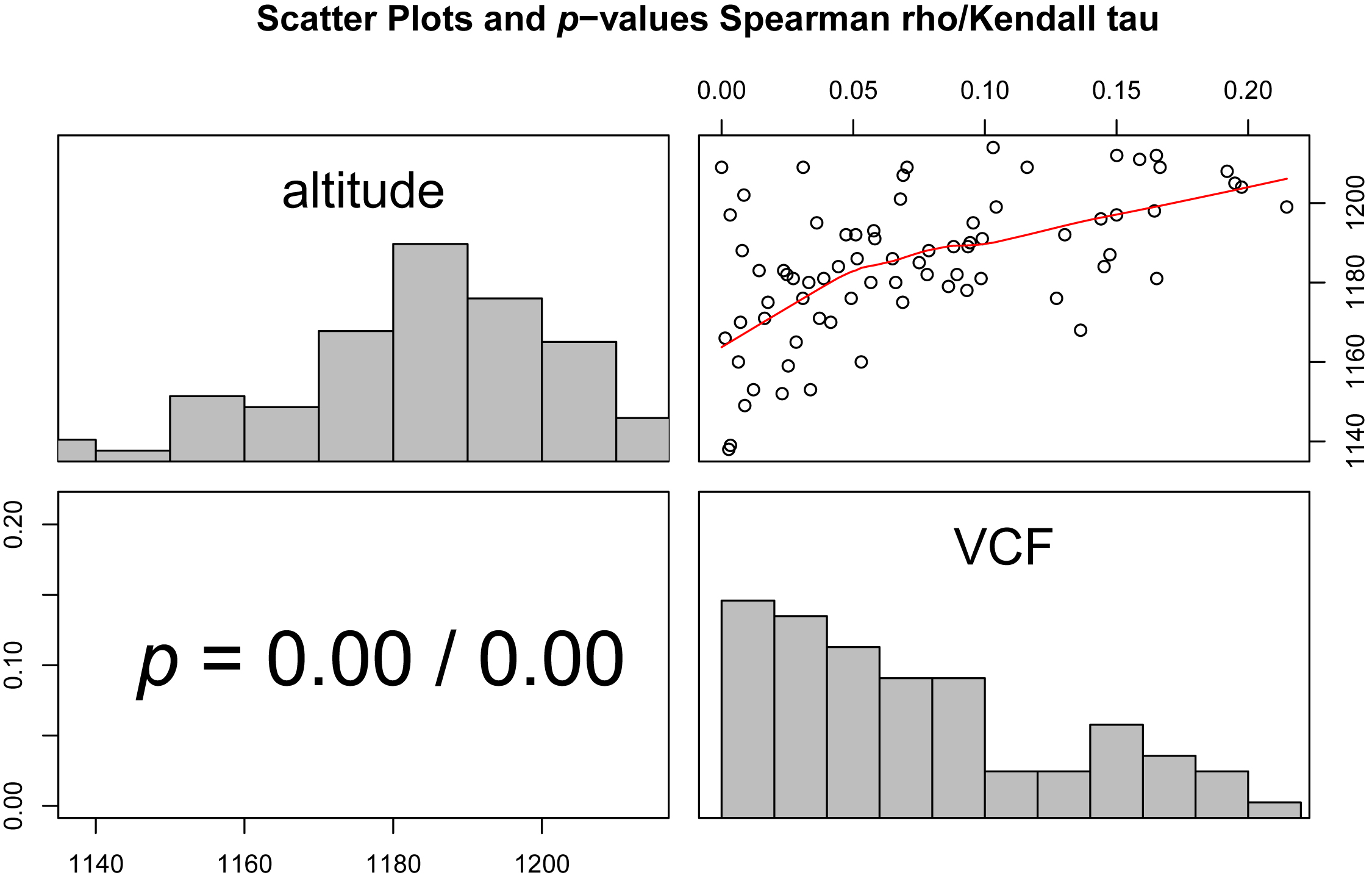

Supplement: Supplementary file 5 — Figure S2C [file ECE3-10-13260-s005.jpg]

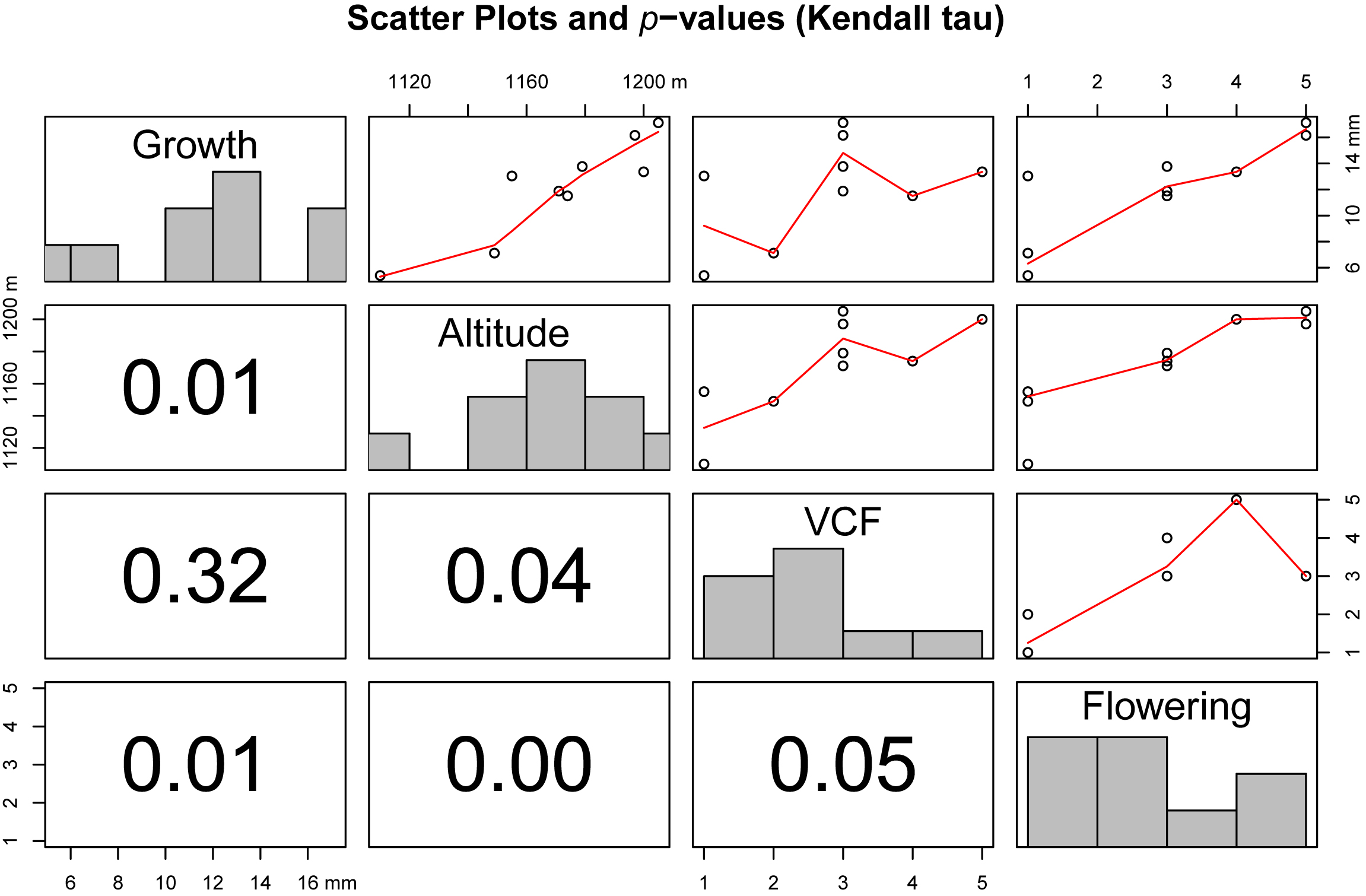

Supplement: Supplementary file 6 — Figure S3 [file ECE3-10-13260-s006.jpg]
