# Supplementary material for: Vegetation growth and landscape genetics of Tillandsia lomas at their dry limits in the Atacama Desert show fine‐scale response to environmental parameters
Source: Ecol Evol. 2020 Oct 28;10(23):13260–74. doi: 10.1002/ece3.6924 (PMC7713976; doi:10.1002/ece3.6924)
Supplement: Supplementary file 7 — Figures S1–S3 [file ECE3-10-13260-s007.docx]

**Appendix**

Supporting Information (SI) S1 to S3

S1: In addition to Fig. 7A and C from the main document, Fig. S1A and B show the regression line calculated for all four growing seasons and all nine plots separately. Accordingly, there are 36 data points (compared to nine in Fig. 7A and C).

Fig. S1A. Scatterplot of mean growth per month versus altitude. The regression line is indicated. Growth is plotted for any of the four growing periods.

Fig. S1B. Scatterplot of mean growth per month versus vegetation coverage fraction. The regression line is indicated. Growth is plotted for any of the four growing periods.

S2: The following scatterplots refer to landscape comparisons of genetic diversity, altitude, and vegetation cover fraction (VCF) and correspond to Table 1 of the main document.


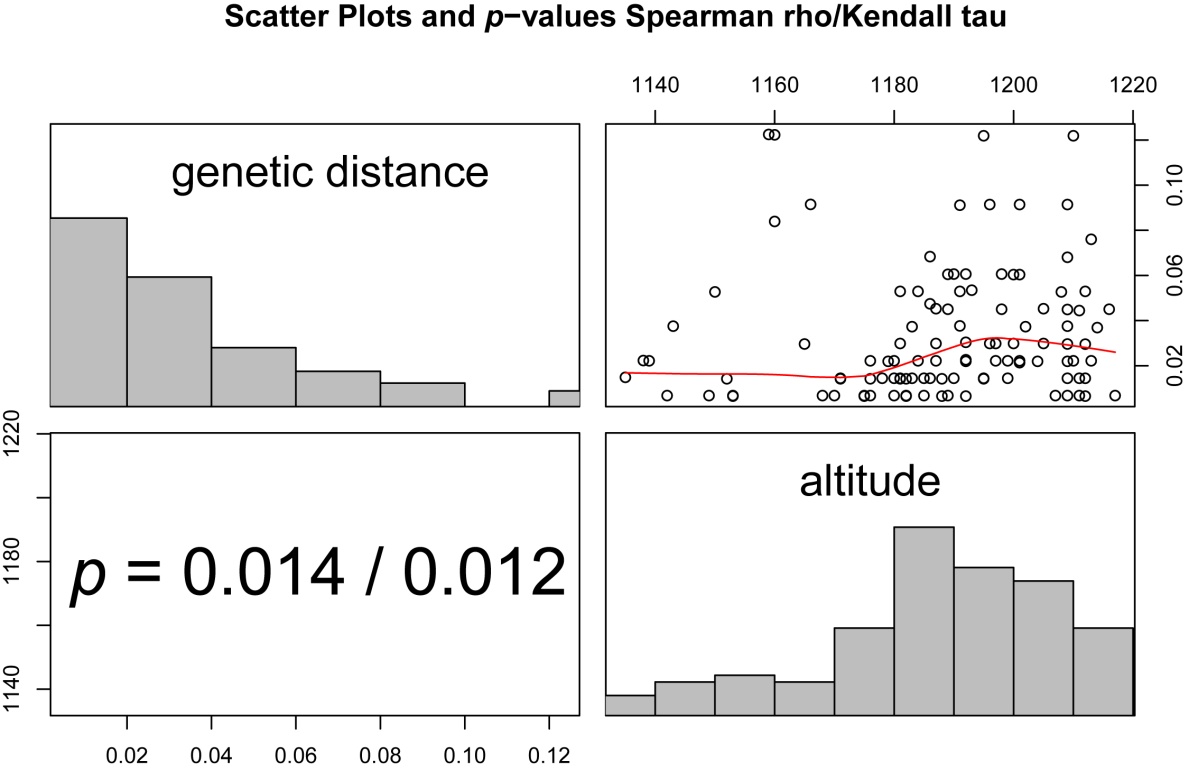


Fig. S2A. Scatterplot of genetic distance (results from AIS, Alleles in Space) versus altitude (3D-landscape model) and *p*-values from respective Spearman rho and Kendall tau.


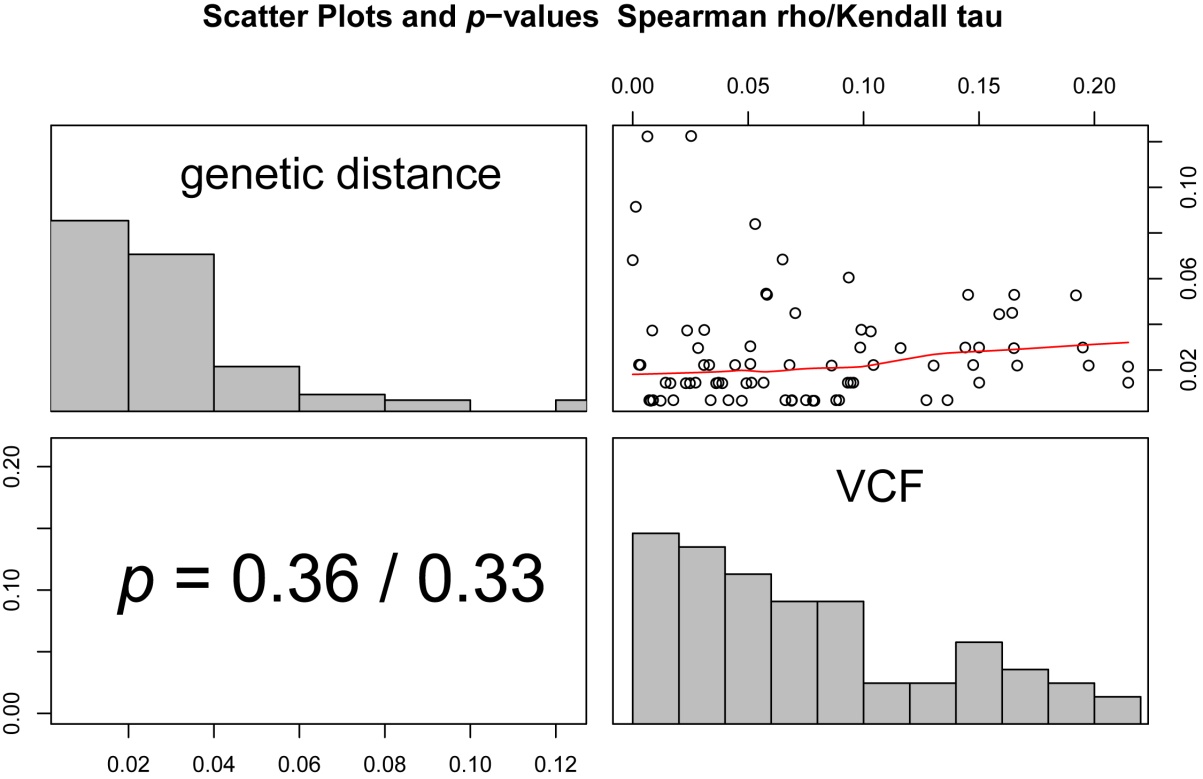


Fig. S2B. Scatterplot of genetic distance (results from AIS) versus vegetation cover fraction (VCF) and *p*-values from respective Spearman rho and Kendall tau.


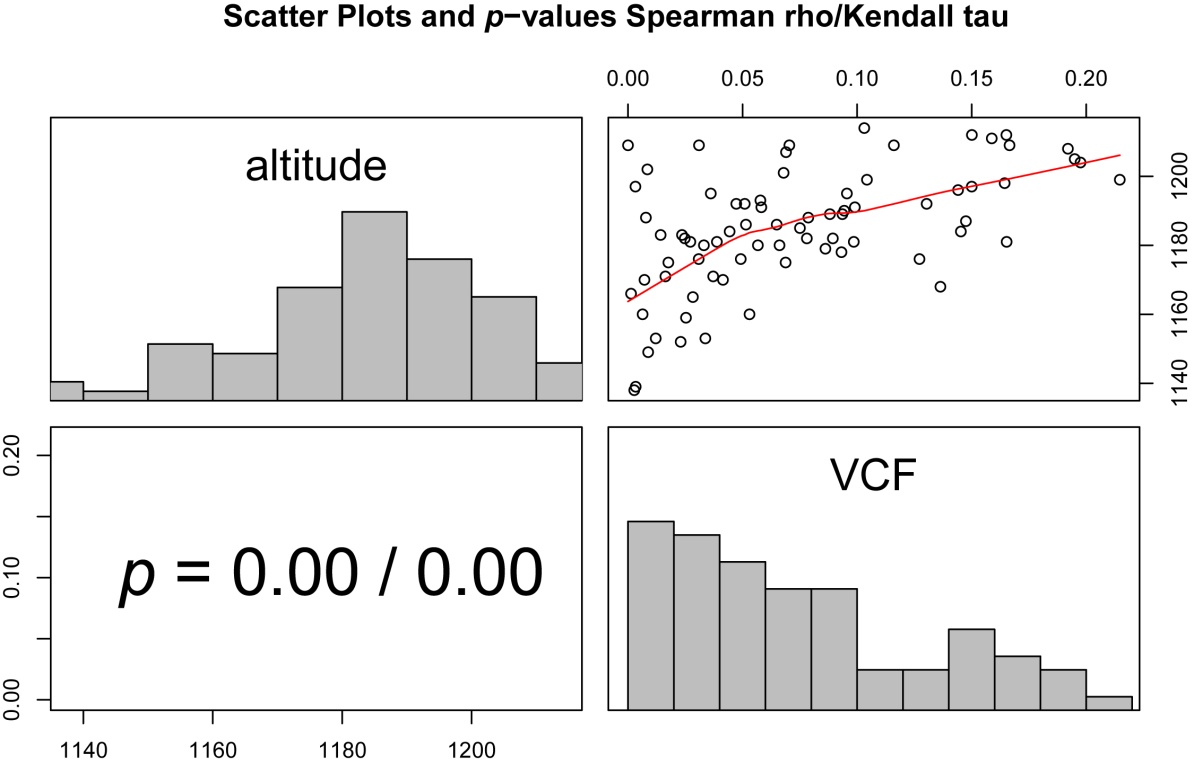


Fig. S2C. Scatterplot of altitude versus vegetation cover fraction and *p*-values from respective Spearman rho and Kendall tau.

S3. Summary scatter plots for individual study plots.


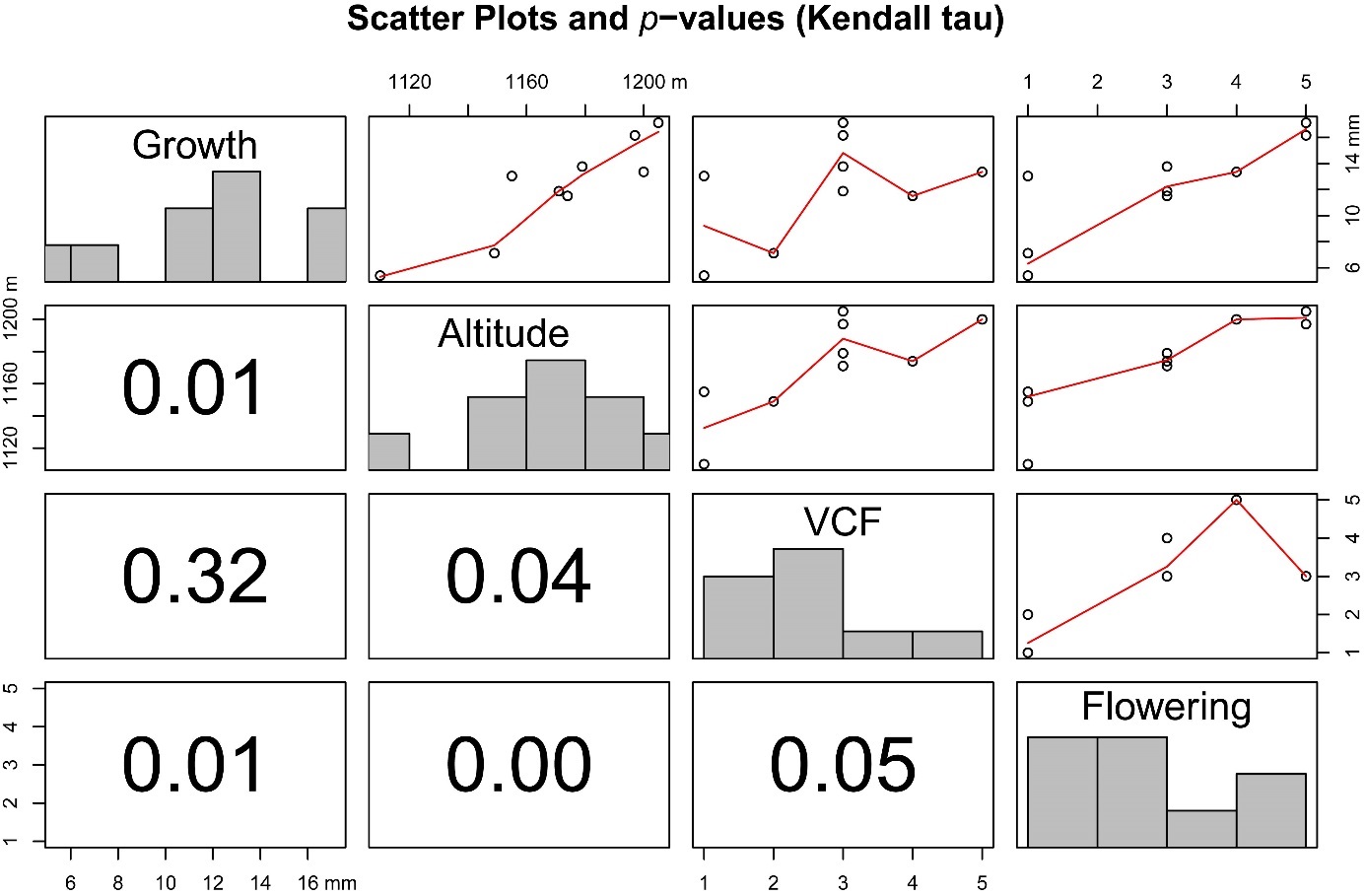


Fig. S3. Scatterplots of the comparisons encompassing growth per year, altitude, vegetation cover fraction, and flower density from the comparison among the nine study plots at Oyarbide. The graphs correspond to Table 3 from the main document.
